# DNA methylation associates with regional tau pathology beyond age and APOE

**DOI:** 10.1002/alz.092539

**Published:** 2025-01-03

**Authors:** David Goldberg, Anil R Wadhwani, David A Wolk, Eddie B Lee, David A. Bennett, Philip L. De Jager, Julie A. Schneider, Wanding Zhou, Corey T McMillan

**Affiliations:** ^1^ University of Pennsylvania, Philadelphia, PA USA; ^2^ Rush University Medical Center, Chicago, IL USA; ^3^ Columbia University Medical Center, New York, NY USA; ^4^ Children’s Hospital of Philadelphia, Philadelphia, PA USA; ^5^ Penn Frontotemporal Degeneration Center, Department of Neurology, Perelman School of Medicine, University of Pennsylvania, Philadelphia, PA USA

## Abstract

**Background:**

In AD, neurofibrillary tangles (NFTs) develop earliest in the limbic system before spreading to neocortical areas. When accounting for covariates of AD pathology, such as age and APOE, there remains interindividual variation in NFT spread in the brain. We therefore used a machine‐learning approach to investigate whether age‐independent DNA methylation (DNAm) changes in brain associate with histopathological differences in AD.

**Method:**

We analyzed DNAm data obtained using the HM450 array and preprocessed using the R package SeSAMe from dorsolateral prefrontal cortex of Religious Orders Study and Memory and Aging Project (ROSMAP; N = 707) participants. We trained age‐adjusted elastic net regression models to predict NFTs in middle frontal cortex, hippocampus, and a summary score from 5 regions (“total”) using 5‐fold cross‐validation. To determine if DNAm affords additional predictive power, we calculated the variance and the error of regression models that included age, sex, and APOE with or without DNAm from elastic net‐identified CpGs.

**Result:**

Age‐adjusted elastic net models of frontal cortex DNAm associated with total (cor = 0.25, p = 0.0033) and midfrontal (cor = 0.26, p = 0.0017), but not with hippocampal (cor = 0.08, p = 0.34) NFTs. Multiple regression models that included DNAm outperformed models that predicted using age, sex and APOE alone (+ DNAm: total: adjR^2^ = 0.682, rmse = 0.175; midfrontal: adjR^2^ = 0.594, rmse = 0.479) (‐ DNAm: total: adjR^2^ = 0.682, rmse = 0.175; midfrontal: adjR^2^ = 0.594, rmse = 0.479). Frontal cortex DNAm was least associated with hippocampal NFTs (adjR^2^ = 0.193, rmse = 1.20).

**Conclusion:**

DNAm provides an additional mechanism to account for variance in NFTs amongst aging individuals. However, frontal cortex DNAm is more associated with frontal cortex NFTs than hippocampal NFTs. This finding suggests that region‐specific DNAm may support drivers of p‐tau spread between regions. Future research is necessary to determine whether DNAm differences across AD are causal or consequential.

